# *Pentatrichomonas hominis* Infection Induces Chronic Intestinal Inflammation in Immunocompetent Mice

**DOI:** 10.1155/tbed/8820836

**Published:** 2025-12-13

**Authors:** Yao Rong, Yidan Cheng, Hongbo Zhang, Xichen Zhang, Yu Zheng, Jianhua Li, Pengtao Gong, Xiaocen Wang, Xin Li, Nan Zhang

**Affiliations:** ^1^ State Key Laboratory for Diagnosis and Treatment of Severe Zoonotic Infectious Diseases, Key Laboratory for Zoonosis Research of the Ministry of Education, Institute of Zoonosis, College of Veterinary Medicine, Jilin University, Changchun, 130062, China, jlu.edu.cn

**Keywords:** gut microbiota, inflammation, pathogen, pathogenicity, *Pentatrichomonas hominis*

## Abstract

*Pentatrichomonas hominis* (*P. hominis*) has traditionally been regarded as a commensal or opportunistic inhabitant of the intestine. However, a growing number of studies have identified this protozoan as the sole pathogen in cases of intestinal disorders, including diarrhea, in both humans and animals, suggesting it may be a neglected zoonotic pathogen. To investigate the pathogenicity of *P. hominis*, we here systematically evaluated its effects on healthy immunocompetent BALB/c mice. Our findings revealed that infection with *P. hominis* induced injury in the large intestine, marked by inflammatory cell infiltration, epithelial cell necrosis, and intestinal mucosal sloughing. These pathological changes persisted and worsened throughout the 90‐day observation period. Furthermore, infection appeared to disrupt goblet cell maturation or secretion, as indicated by increased periodic acid–Schiff (PAS) staining alongside decreased MUC2 production. Elevated levels of pro‐inflammatory cytokines were also detected in both intestinal lavage fluid and serum. Finally, *P. hominis* infection altered the composition of the gut microbiota, increasing both its richness and diversity. Notably, it raised the relative abundance of the inflammation‐associated genus *Desulfovibrio*, while reducing the abundance of beneficial bacteria, such as *Akkermansia*, *Roseburia*, and *Lactobacillus*. Collectively, these results provide compelling evidence that *P. hominis* acts as a zoonotic pathogen capable of inducing chronic intestinal inflammation. Therefore, the clinical significance of *P. hominis* infection warrants attention.

## 1. Introduction

*Pentatrichomonas hominis* (*P. hominis*) is a flagellated protozoan of the trichomonad group, characterized by the presence of four anterior flagella, one independent flagellum, and one recurrent flagellum [1, 2]. It primarily colonizes warm, moist, and anaerobic regions of the large intestine in a broad range of hosts, including humans, dogs, cats, rats, and pigs [3, 4]. The life cycle of *P. hominis* is relatively simple. The trophozoite represents its predominant form, though it cannot survive for extended period in the environment [5]. In contrast, *P. hominis* can also form pseudocyst, which enable it to endure several days under unfavorable conditions [6, 7]. Transmission of *P. hominis* occurs directly between hosts, most likely via the fecal–oral route through ingestion of contaminated food or water [1, 8, 9]. Molecular analyses of 18S rRNA sequences from human isolates have shown that some are identical to those obtained from animals, such as dogs and cats, supporting the zoonotic potential of *P. hominis* [10, 11].

*P. hominis* was initially regarded as a nonpathogenic commensal in the intestine [12]. However, its pathogenic role has become a subject of debate, as mounting evidence has identified this protozoan in cases of diarrhea in humans and animals, as well as in patients with irritable bowel syndrome (IBS) [6, 8, 9, 13]. Epidemiological studies further indicate a higher prevalence of *P. hominis* infection in diarrhea hosts. For instance, a study in East China reported an infection rate of 50.6% (43/85) in dogs with diarrhea, significantly greater than the 24.3% (56/230) observed in nondiarrheal dogs [14]. Similarly, a coproscopic survey in Egypt revealed a strong association between *P. hominis* infection and diarrhea and abdominal pain in school‐aged children [15]. Such findings suggest that *P. hominis* may act as an etiological agent of intestinal symptoms in both humans and animals. Additionally, *P. hominis* infection has been linked to several systemic conditions, including systemic lupus erythematosus, rheumatoid arthritis, and gastrointestinal cancers in humans, as well as blackhead disease in turkeys [10, 16–18]. Although a recent in vitro study demonstrated the potential pathogenic effects of *P. hominis* on porchine intestinal epithelial cells (IPEC‐J2) [19], the precise pathogenic role of this protozoan and its underlying mechanisms in human and animal health remain unclear and warrant further investigation.

Intestinal protozoans, together with bacteria, viruses, and other eukaryotes, constitute the gut microbiota [20]. These diverse microorganisms interact with each other and with the host, forming a complex internal ecosystem [21]. A dynamic yet stable gut microbiota is widely recognized as beneficial to host health, whereas an imbalance in this ecosystem—known as dysbiosis—can compromise host wellbeing and has been strongly linked to gastrointestinal disorders, such as IBS, inflammatory bowel disease (IBD), and colorectal cancer (CRC) [21, 22]. The interplay between intestinal protozoans and gut bacteria is complex and remains poorly understood. Emerging evidence suggests that intestinal protozoans can alter the diversity and abundance of gut bacteria, which in turn can influence protozoan proliferation and virulence. This reciprocal interaction may thereby shape the progression of gastrointestinal diseases [23, 24]. A recent study in foxes reported lower diversity and richness of the gut microbiota in *P. hominis*‐positive individuals compared to *P. hominis*‐negative ones [25]. However, it remains unclear whether these microbial shifts are a direct consequence of *P. hominis* infection. In our previous work, we observed increased diversity and relative abundance of gut bacteria in CRC patients relative to healthy controls [26]. Notably, the relative abundance of several cancer‐associated bacteria—including *Flavonifractor* sp., *Lachnoclostridium* sp., and the *Ruminococcus gnavus* group—was significantly higher in CRC patients coinfected with *P. hominis* than in those without the protozoan [26]. Nevertheless, it is still uncertain whether the observed changes in cancer‐associated gut bacteria are indeed driven by *P. hominis* infection, and if so, whether such alterations contribute to the pathogenesis of CRC.

Given that the pathogenic role of *P. hominis* remains incompletely understood, we conducted a comprehensive analysis of its impacts on the gastrointestinal tract of healthy immunocompetent BALB/c mice. Our findings demonstrate that *P. hominis* infection induces chronic intestinal inflammation and alters the diversity and composition of the gut microbiota. Consequently, the clinical significance and prevalence of this infection warrant greater attention.

## 2. Materials and Methods

### 2.1. Ethics Statement

All animal experiments conducted in this study were approved by the Institutional Animal Care and Use Committee of Jilin University (IACUC Permit Number: SY202201103) and were carried out in accordance with Regulations for the Administration of Affairs Concerning Experimental Animals approved by the State Council of People’s Republic of China.

### 2.2. *P. hominis* Culture, Animal Use and Sample Collection

The *P. hominis* ATCC3000 strain was cultured in modified TYI‐S‐33 medium at 37°C, as previously described [27].

Wild‐type female immunocompetent BALB/c mice (4 weeks of age) were purchased from Beijing Vital River Laboratory Animal Technology Co., Ltd (Beijing, China) and maintained under specific pathogen‐free (SPF) conditions. After 1 week of acclimation, the mice were randomly assigned into groups (*n* = 5 per group) with comparable average body weight.

To determine the minimum infective dose of trophozoites, mice were orally inoculated with 1, 10, 1 × 10^2^, 1 × 10^3^, 1 × 10^4^, 1 × 10^5^, 1 × 10^6^, or 1 × 10^7^ trophozoites via gavage. For subsequent experiments, a dose of 1 × 10^6^ trophozoites was administrated orally. Control mice received an equal volume of PBS by oral gavage.

Following protozoan inoculation, all animals were housed individually. Fresh fecal samples were collected at 7 or 90 days post infection (dpi) and stored at −80°C. Blood samples were collected from retro‐orbital venous plexus, and serum was obtained by centrifuging at 3000 r/min for 15 min and at 4°C. Mice were sacrificed at 15, 30, 60, or 90 dpi. Major organs and tissue samples were collected and stored at −20°C. Intestinal lavage fluid was collected by rinsing the intestinal lumen with PBS.

### 2.3. Nested PCR

Following *P. hominis* infection, fecal samples were collected from the mice, and genomic DNA was extracted using TIANamp Stool DNA Kit (TIANGEN, China) in accordance with the manufacturer’s instructions. Nested PCR was subsequently performed as previously described [28].

### 2.4. Hematoxylin and Eosin (H&E) Staining and Pathological Evaluation

Tissue sections were fixed in 10% formaldehyde solution and embedded in paraffin. The embedded tissues were then sectioned, deparaffinized, and stained with H&E following standard protocols. Pathological examination was performed using an Olympus light microscope (Japan).

Pathological scoring was conducted in a blinded manner by an experienced pathologist, based on the assessment of four parameters as previously described [29]: inflammatory cell infiltration (0–4), extent of inflammation (0–3), crypt damage (0–4), and percent involvement (0–4). The total pathological score, ranging from 0 to 15, was calculated as the sum of these four sub‐scores.

### 2.5. Periodic Acid–Schiff (PAS) Staining

Paraffin‐embedded cecum and colon sections were deparaffinized using xylene and rehydrated through a graded ethanol series (100%, 100%, and 75%). The sections were then sequentially stained as follows: with PAS solution B (0.5% periodic acid) for 10–15 min, followed by PAS solution A (Schiff solution) for 25–30 min in the dark, and finally counterstained with PAS solution C (hematoxylin) for 30 s. Thereafter, the sections were dehydrated through an ethanol series, cleared in xylene, and sealed with neutral gum. Images were acquired using an Olympus light microscope (Japan). Goblet cells were identified as brown or deep purple granules and were quantified blindly by researchers in triplicate.

### 2.6. Immunohistochemistry

Paraffin‐embedded cecum and colon sections were deparaffinized and subjected to antigen retrieval by boiling in EDTA buffer (pH 9.0) for 20 min. Endogenous peroxidase activity was then blocked by treating the sections with 3% hydrogen peroxide for 25 min in the dark. Subsequently, nonspecific binding sites were blocked using rabbit serum at room temperature for 30 min, The sections were incubated overnight at 4°C with rabbit anti‐MUC2 antibody (Servicebio, China) at a dilution of 1:1000, followed by a 50‐min incubation at room temperature with horseradish peroxidase (HRP)‐conjugated anti‐rabbit secondary antibody (Servicebio, China) at 1:200 dilution. Finally, staining was developed using 3,3’‐diamino‐benzidine (DAB), and nuclei were counterstained with hematoxylin. Images were acquired using an Olympus microscope (Japan), and fluorescence intensity was quantified using Image J software.

### 2.7. Enzyme‐Linked Immunosorbent Assay (ELISA)

The levels of IL‐6, TNF‐α, and IFN‐γ were measured using corresponding Mouse ELISA Kits (Jianglaibio, China) according to the manufacturer’s instructions. Prior to assay, serum samples were diluted five fold and intestinal lavage fluid samples were diluted twofold.

### 2.8. TUNEL Assay

Paraffin‐embedded tissue sections were deparaffinized using xylene and rehydrated through a graded ethanol series. The sections were then treated with protease K working solution to cover the tissue and incubated at 37°C for 20 min. After washing, the equilibration buffer was applied to cover the samples and incubated at room temperature for 10 min. The TdT enzyme, dUTP, and reaction buffer were mixed at a ratio of 1:5:50 to prepare the labeling reaction mixture. This mixture was applied to cover the tissue sections, followed by incubation at 37°C for 1 h in a humidified chamber. The sections were then stained with DAPI solution and incubated at room temperature for 10 min in the dark. Finally, the slides were mounted with antifade mounting medium. Images were acquired using a fluorescence microscope (Nikon, Japan) and fluorescence intensity was quantified using ImageJ software.

### 2.9. Immunofluorescence

For Ki67 staining, paraffin‐embedded tissue sections were deparaffinized in xylene and rehydrated through a graded ethanol series. The sections were then blocked with 3% bovine serum albumin (BSA) for 30 min at room temperature, followed by incubation overnight at 4°C with rabbit anti‐Ki67 primary antibody (Servicebio, China) at a dilution of 1:500. After washing, the sections were incubated with a fluorescence‐conjugated anti‐rabbit secondary antibody (Servicebio, China) at 1:300 dilution for 50 min at room temperature in the dark. Nuclei were counterstained with DAPI for 10 min. Finally, AutoFluo Quencher B was applied for 5 min to reduce background autofluorescence. All mages were acquired using a fluorescence microscope (Nikon, Japan) and fluorescence intensity was quantified using ImageJ software.

### 2.10. DNA Extraction, 16 S rRNA Sequencing, and Bioinformatic Analysis

Total genomic DNA was extracted from fecal samples using the cetyltrimethylammonium bromide (CTAB) method according to the manufacturer’s instructions. DNA quality and concentration were assessed by agarose gel electrophoresis and ultraviolet spectrophotometer, respectively. The V3–V4 hypervariable regions of the bacterial 16 S rRNA gene were amplified by PCR using extracted DNA and universal primers 341F (5′‐CCTACGGGNGGCWGCAG‐3′) and 805R (5′‐GACTACHVGGGTATCTAATCC‐3′), each tagged with unique barcodes. The PCR products were evaluated by agarose gel electrophoresis, purified with AMPure XT beads (Beckman Coulter Genomics, USA), and quantified using a Qubit fluorometer (Invitrogen, USA). The purified amplicons were pooled to construct a sequencing library, which was assessed for size and quantity using an Agilent 2100 Bioanalyzer (Agilent, USA) and the Kapa Library Quantification Kit (Kapa Biosciences, USA), respectively. Sequencing was performed on the Illumina NovaSeq PE250 platform with a NovaSeq 6000 SP Reagent Kit (Illumina, USA) to generate 2 × 250 bp paired‐end reads. Raw paired‐end reads were demultiplexed based on sample‐specific barcodes, and the primers and barcodes were trimmed using Cutadapt (1.9). Reads were then assembled using FLASH (v1.2.8) and quality filtering was performed with fqtrim (v0.94) to generate high‐quality clean tags. Chimeric sequences were identified and removed using Vsearch (v2.3.4) [30]. Dereplication and resolution of amplicon sequence variants (ASVs) were conducted using DADA2, producing a feature table and feature sequences. Alpha and beta diversity analyses were performed in QIIME2 [31]. Representative sequences were aligned by BLAST and taxonomically annotated against the SILVA database (v138) [32].

### 2.11. Statistical Analysis

All statistical analyses were performed using GraphPad Prism software (version 8.0). Data derived from at least three independent biological replicates are presented as the mean ± SEM. Details of the specific statistical tests applied in each experiment are provided in the corresponding figure legends. A *p*‐value of less than 0.05 was considered statistically significant and significance levels are denoted as follows:  ^∗^ *p* < 0.05,  ^∗∗^ *p* < 0.01,and  ^∗∗∗^ *p* < 0.001.

## 3. Results

### 3.1. *P. hominis* Infection Induces Injury in the Large Intestine of Immunocompetent Mice

To evaluate the impact of *P. hominis* infection on immunocompetent mice, we first determined the lowest infectious dose of trophozoites (see Section 2 and Figure 1A for the experimental design). Trophozoites were only detected in the stools of mice inoculated with 1 × 10^6^ or 1 × 10^7^ trophozoites (Figure S1A). Consistent with this, nested PCR also confirmed the presence of *P. hominis* DNA exclusively in stool samples from mice inoculated with 1 × 10^6^ or 1 × 10^7^ trophozoites (Figure S1B). Thus, the minimum infectious dose for BALB/c mice was established as 1 × 10^6^ trophozoites.

Figure 1
*P. hominis* infection induces intestinal injury in the cecum and colon of immunocompetent BALB/c mice. (A) Experimental design and parasite morphology. Schematics of the experimental design and morphological structure of *P. hominis* (dashed rectangle). Wild‐type BALB/c mice were inoculated with different doses of trophozoites on day 0. Fecal and tissue samples were collected at indicated time points for parasite detection and pathological evaluation, respectively. AF, anterior flagella; IF, independent flagellum; RF, recurrent flagellum; UM, undulating membrane. Plus symbol within a circle indicates sample collection. (B) Body weight changes. Body weight changes of mice (*n* = 5) after inoculation with a low‐dose (1 × 10^6^ trophozoites) or a high‐dose (1 × 10^7^ trophozoites) of *P. hominis*. (C) Histopathological analysis. Representative H&E‐stained sections of cecal and colon tissues from each group were shown at high magnification (100 × ) and low magnification (40 × ; inserts). Large black arrows indicate inflammatory cell infiltration; blue arrows denote attenuation of the muscular layer; small arrows highlight cellular debris. Ctrl: control; dpi: days post infection. Scale bars: 100 μm (white) and 200 μm (black). Figure S2 for additional details. (D) Histological scoring. Histological scores of the cecum and colon tissues from the indicated groups (*n* = 3). Data are presented as mean ± SEM. Statistical analyses were determined by two‐way ANOVA (B) or one‐way ANOVA (D), with significance levels denoted as  ^∗^ *p* < 0.05,  ^∗∗^ *p* < 0.01,  ^∗∗∗^ *p* < 0.001; ns, not significant.

**Figure:**
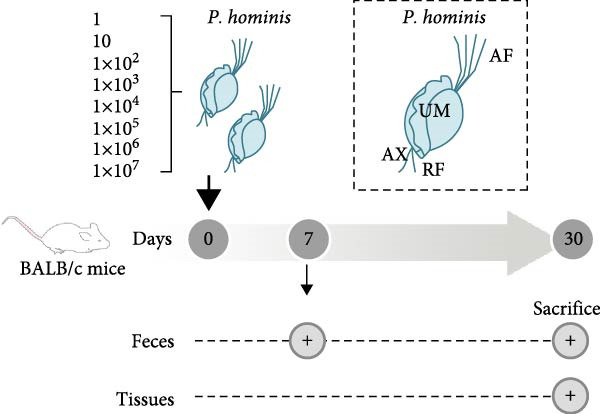
(A)

**Figure:**
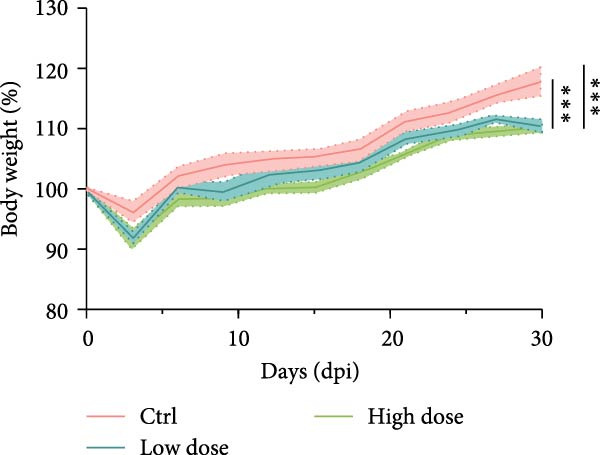
(B)

**Figure:**
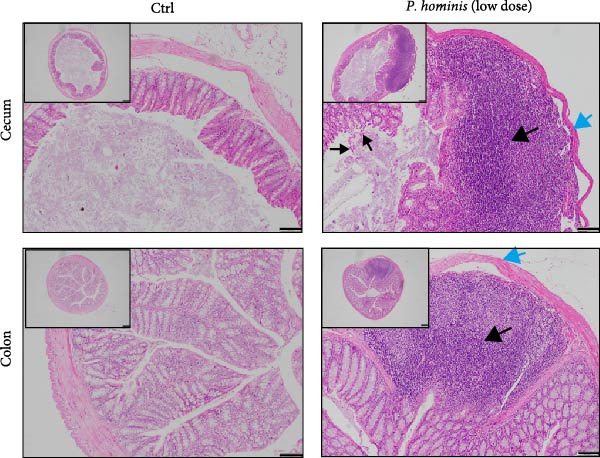
(C)

**Figure:**
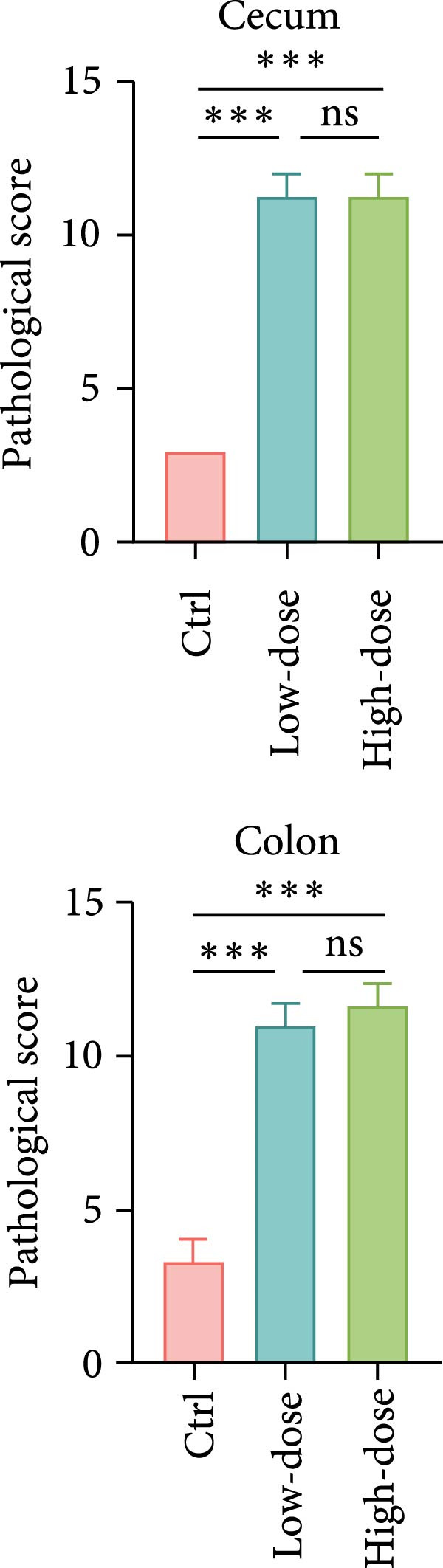
(D)

On day 30 postinoculation, mice were euthanized, and cecum and colon tissues were collected from both infected and uninfected groups for fixation and H&E staining. Over the 30‐day period, mice infected with 1 × 10^6^ (low‐dose) or 1 × 10^7^ (high‐dose) trophozoites exhibited slower body weight gain compared to uninfected controls (*p*  < 0.05; Figure 1B), suggesting that *P. hominis* infection may impair intestinal nutrient absorption in immunocompetent BALB/c mice. Furthermore, H&E staining revealed pathological damage in both the cecum and colon of infected mice, characterized by inflammatory cell infiltration (predominantly neutrophils) in the lamina propria and muscularis mucosae, epithelial cell necrosis, sloughing of the intestinal mucosa at the villus tips, and thinning of the muscular layer (Figure 1C and Figure S2). Consistent with these observations, histological scores of the cecum and colon were significantly higher in mice infected with either low‐dose or high‐dose than in uninfected mice (*p*  < 0.001; Figure 1D). No significant difference in histopathological severity was observed between the two infected groups (*p* > 0.05; Figure 1D). Given that 10^6^ trophozoites dose successfully established a detectable infection and induced significant large intestinal injury in immunocompetent BALB/c mice without mortality, it was selected as the standard dose above the detection limit.

Taken together, these results demonstrate that *P. hominis* infection causes significant injury in the large intestine of immunocompetent BALB/c mice, with a minimum infectious dose of 1 × 10^6^ trophozoites.

### 3.2. *P. hominis* Infection Induces Sustained and Progressive Injury in the Large Intestine

To evaluate the progressive effects of *P. hominis* infection, we monitored infected mice over a 90‐day period (see Material and Methods and Figure 2A for the experimental design). Throughout the observation period, infected mice exhibited significantly slower body weight gain compared to uninfected controls (*p*  < 0.001; Figure 2B). Moreover, the large intestine of infected mice was significantly shorter than that of controls at 90 dpi (*p*  < 0.01, Figure 2C).

Figure 2The pathogenic effects of *P. hominis* infection persist over time in BALB/c mice. (A) Experimental design. BALB/c mice were infected with a low‐dose of trophozoites on day 0. Fecal and tissue samples were collected at the indicated time points for 16 S rRNA sequencing and pathological analysis, respectively. (B) Body weight changes. *P. hominis*‐infected mice exhibited slower weight gain compared to uninfected controls over the 90‐day period (*n* = 10). (C) Large intestine length. Left: quantification of large intestine length in the indicated groups at 15, 30, 60, and 90 days post infection (dpi; *n* = 5). Right: macroscopic images of large intestines from the each group at 90 dpi. (D) Histopathological analysis. Representative H&E‐stained sections of cecal and colon tissues from the indicated groups were shown at low magnification (low mag; 100 × ; left) and high magnification (high mag; 400 × ; right; corresponding boxed areas). Lower magnification images (40 × ) are available in Figure S3. Black and blue arrows indicate inflammatory cell infiltration and muscular layer attenuation, respectively. Scale bars: 100 μm (black) and 20 μm (white). (E) Histological scoring. Histological scores of cecum and colon tissues from the indicated mice (*n* = 3). Data are presented as mean ± SEM. Statistical analysis was performed using two‐way ANOVA (B, C left panel and E left panel) or one‐way ANOVA (C right panel and E right panel). Statistical significance is indicated as  ^∗^ *p* < 0.05, ^∗∗^ *p* < 0.01,  ^∗∗∗^ *p* < 0.001; ns, not significant.

**Figure:**
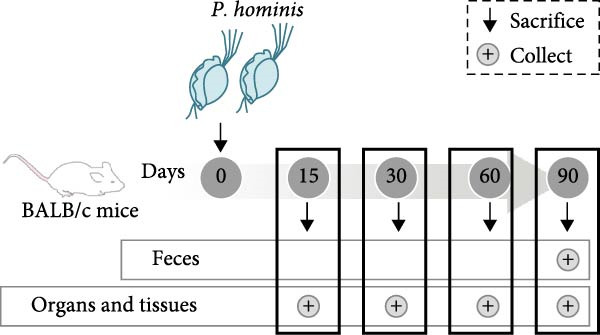
(A)

**Figure:**
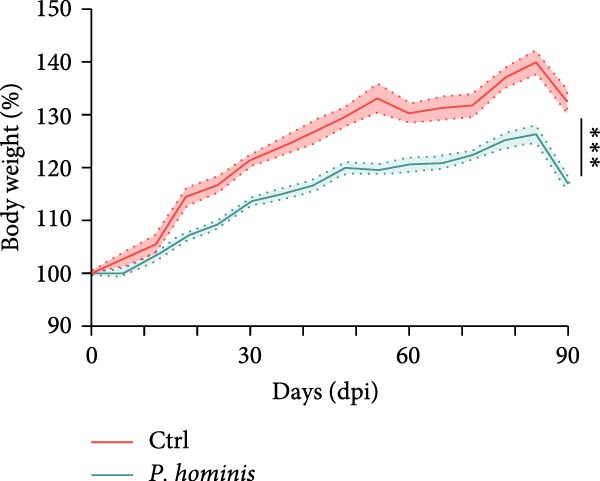
(B)

**Figure:**
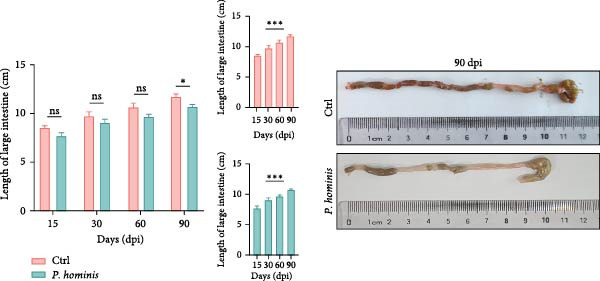
(C)

**Figure:**
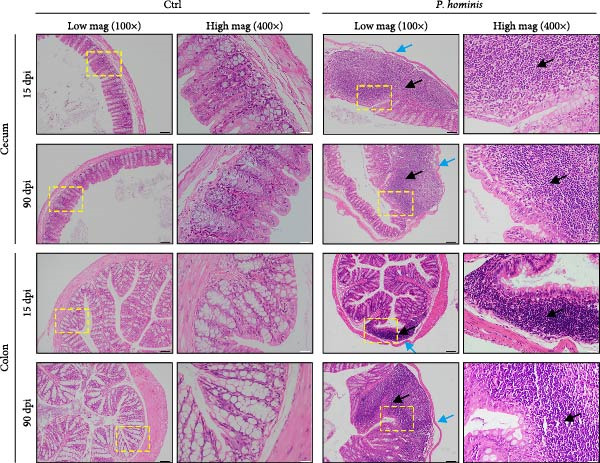
(D)

**Figure:**
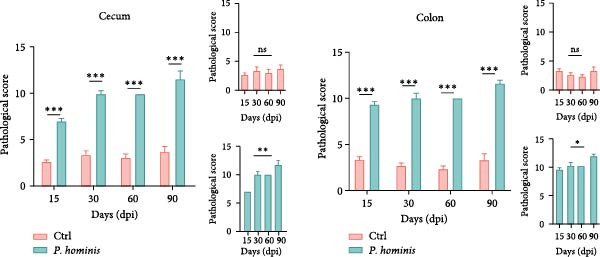
(E)

Histopathological analysis of cecum and colon tissues by H&E staining revealed sustained damage in infected mice. In the cecum, substantial inflammatory cell infiltration, epithelial cell necrosis, mucosal sloughing, and thinning of the muscular layer were observed at all examined time points after inoculation (Figure 2D and Figure S3). Notably, by 90 dpi, *P. hominis* infection led to marked intestinal inflammation, with apparent shortening or loss of intestinal villi in the cecum (Figure 2D and Figure S3). Consistent with these findings, histopathological scores were significantly higher in infected mice than in controls and showed a progressive increase over time (*p*  < 0.01; Figure 2E), indicating a worsening of cecal injury.

Similarly, colon sections from infected mice displayed inflammatory cell infiltration, epithelial necrosis, mucosal sloughing, and muscular layer attenuation at all time points. By 90 dpi, crypt depths and villus heights were notably reduced (Figure 2D and Figure S3). Correspondingly, pathological scores in the colon were significantly elevated in infected mice relative to controls, and increased progressively over the course of infection (*p*  < 0.05; Figure 2E).

Collectively, these results demonstrate that *P. hominis* infection causes erosive and ulcerative lesions in the large intestine, with pathological changes that not only persist but also gradually worsen over time.

### 3.3. *P. hominis* Infection Also Affects the Small Intestine and Lung

In light of the reported association between *P. hominis* infection and small intestinal cancer [10], we assessed the jejunum and ileum of both infected and uninfected mice using H&E staining. The results showed mild but significant pathological damage in both segments of the small intestine following infection. This was characterized by a reduction in microvillus length in the jejunum and ileum, along with infiltration of tissue‐resident inflammatory cells in the ileum (Figure 3A). Consistent with these observations, histopathological scores were significantly higher in the jejunum and ileum of infected mice compared to uninfected controls (Figure 3B).

Figure 3
*P. hominis* infection induces pathological damage in the small intestine. (A) Histopathological analysis. Representative H&E‐stained sections of jejunum and ileum from the indicated groups were shown at low magnification (100× ; left) and high magnification (400×; right), with the higher magnification views corresponding to the boxed regions. Arrows indicate inflammatory cell infiltration. Scale bars: 100 μm (black) and 20 μm (white). (B) Histological scoring. Histological injury scores for jejunum and ileum tissues from the indicated mice. Data are presented as mean ± SEM (*n* = 3). Statistical analysis was performed using two‐way ANOVA (left panel) or one‐way ANOVA (right panel). Statistical significance is indicated as  ^∗^ *p* < 0.05,  ^∗∗^ *p* < 0.01,  ^∗∗∗^ *p* < 0.001; ns, not significant.

**Figure:**
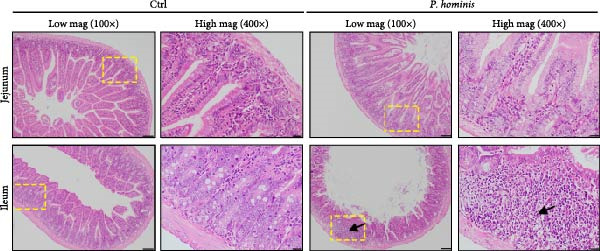
(A)

**Figure:**
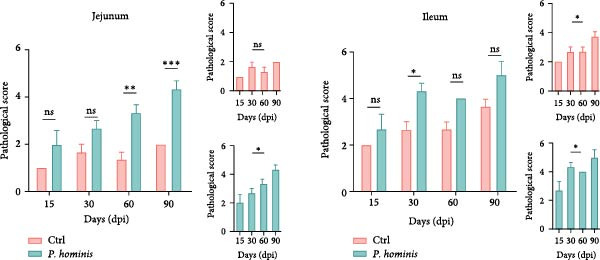
(B)

To further evaluate the systemic effects of *P. hominis* infection, we also evaluated the heart, liver, spleen, kidney, and stomach for any pathological alterations. Although no obvious morphological or histological changes were detected in these organs (Figure S4A,B), we occasionally noted thickening of the alveolar septum in the lungs of infected mice (Figure S4A).

In summary, these results demonstrate that *P. hominis* infection can induce pathological changes not only in the small intestine but also occasionally in the lung, whereas other major parenchymal organs in immunocompetent BALB/c mice remained unaffected under the present experimental conditions.

### 3.4. *P. hominis* Infection Induces Chronic Intestinal Inflammation

Given that abnormal goblet cell counts and altered mucins secretion are known to contribute to intestinal inflammation [33], we performed PAS staining on cecum and colon sections from infected and uninfected mice at 90 dpi to assess goblet cell levels. Notably, PAS staining intensity was markedly increased in both cecum and colon following *P. hominis* infection (Figure 4A and Figure S5A). In contrast, MUC2 intensity was significantly reduced in these intestinal regions (Figure 4B and Figure S5B).

Figure 4
*P. hominis* infection alters goblet cell function and cytokines profiles. (A) Goblet cell quantification. Representative images of PAS staining (top) and quantitative analysis of goblet cell numbers (bottom) in cecal and colonic sections from the indicated groups (*n* = 3). Scale bars: 20 μm. Figure S5 for more details. (B) MUC2 expression analysis. Representative images of MUC2 histochemical staining (top) and quantitative analysis of relative MUC2 expression intensity (bottom) in cecal and colonic tissues from the indicated mice (*n* = 5). Scale bars: 20 μm. See Figure S5 for additional details. (C–E) Cytokine level measurements. Levels of IL‐6 (C), IFN‐γ (D), and TNF‐α (E) in intestinal lavage fluid and serum were measured by ELISA (*n* = 5). All data are presented as mean ± SEM. Statistical significance was determined by two‐tailed Student’s *t*‐test (A, B) or two‐way ANOVA (C–E). Statistical significance is indicated as  ^∗^ *p* < 0.05,  ^∗∗^ *p* < 0.01,  ^∗∗∗^ *p* < 0.001; ns, not significant.

**Figure:**
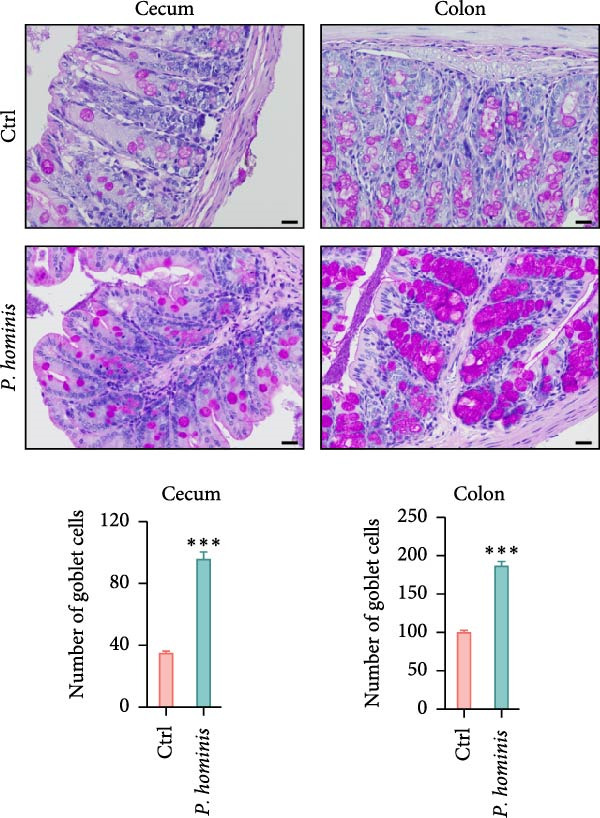
(A)

**Figure:**
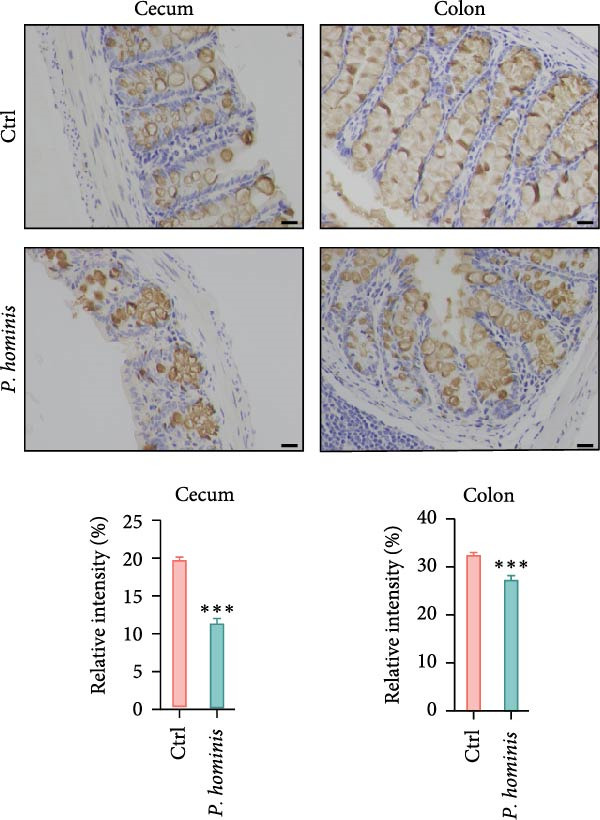
(B)

**Figure:**
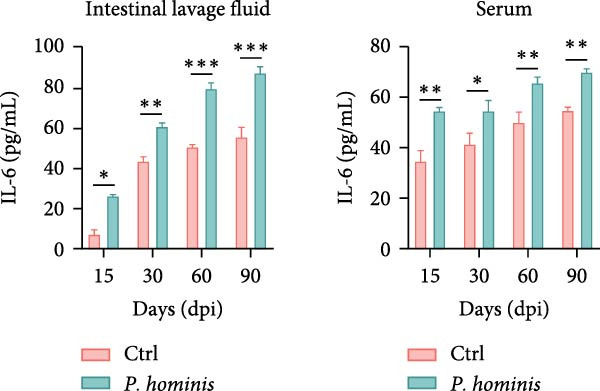
(C)

**Figure:**
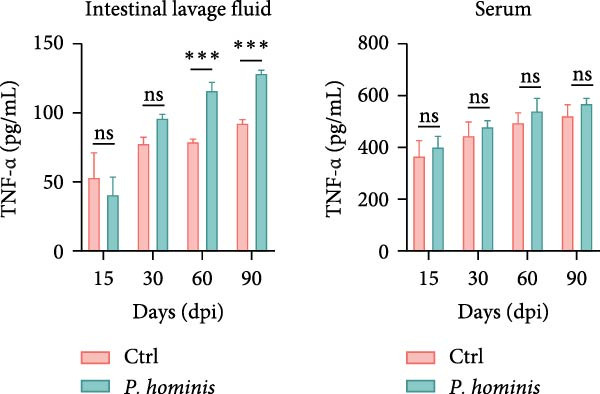
(D)

**Figure:**
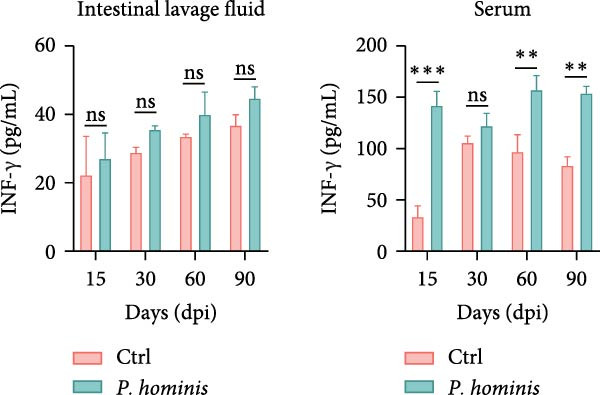
(E)

To further investigate whether *P. hominis* infection triggers intestinal inflammation, we measured the concentrations of pro‐inflammatory cytokines (IL‐6, TNF‐α, and IFN‐γ) on both intestinal tissues and serum using ELISA. The results revealed that IL‐6 levels were significantly elevated in the intestine and serum as early as 15 dpi and remained high throughout the 90‐day period (Figure 4C). TNF‐α levels were increased in the intestine starting at 60 dpi and persisted until 90 dpi, though no significant difference was observed in serum compared to controls (Figure 4D). Furthermore, *P. hominis* infection also led to elevated IFN‐γ concentrations in the serum over the 90‐day period, whereas no notable change was detected in intestinal tissues (Figure 4E).

Collectively, these findings suggest that *P. hominis* infection may disrupt goblet cell maturation or mucin secretion, while concurrently elevating pro‐inflammatory cytokine levels, thereby contributing to chronic intestinal inflammation.

### 3.5. Intestinal Epithelial Cell Homeostasis is Disrupted by *P. hominis* Infection

The tight regulation of epithelial turnover is essential for intestinal physiology, and its dysregulation has been linked to human diseases, such as IBD and cancer [34]. To evaluate whether *P. hominis* infection affects intestinal epithelial cell turnover, we collected cecum and colon tissues from infected and uninfected mice at 90 dpi, and performed proliferation and apoptosis analyses. We first assessed epithelial cell proliferation by Ki67 staining of cecum and colon sections. The results revealed abnormal epithelial hyperplasia in the crypts of both intestinal regions following infection (Figure 5A). In addition, TUNEL assays showed a marked increase in apoptotic epithelial cells at the villus tips of the cecum and colon (Figure 5B). Together, these findings indicate that *P. hominis* infection disrupts intestinal epithelial cell homeostasis and accelerates epithelial cell turnover in the large intestine.

Figure 5
*P. hominis* infection disrupts epithelial cell homeostasis in the cecum and colon. (A) Epithelial cell proliferation. Left: representative immunofluorescence images of Ki67 (red) and DAPI (blue) staining in cecal and colon tissues from infected and uninfected mice at 90 dpi. Right: quantitative analysis of epithelial cell proliferation rates. (B) Epithelial cell apoptosis. Left: representative TUNEL (green) and DAPI (blue) staining in cecal and colon tissues from infected and uninfected mice at 90 dpi. Right: quantitative analysis of apoptotic cell rates. Data are presented as mean ± SEM (*n* = 3). Statistical significance was determined by two‐tailed Student’s *t*‐test ( ^∗∗∗^ *p* < 0.001). Scale bars: 20 μm.

**Figure:**
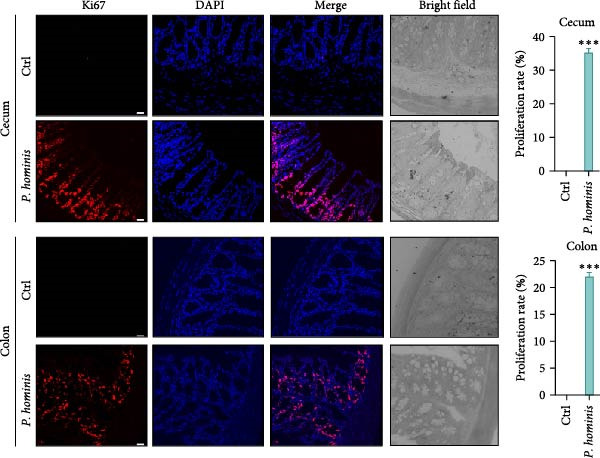
(A)

**Figure:**
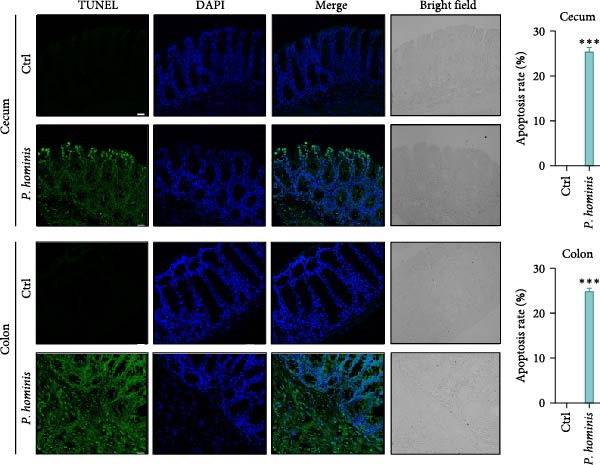
(B)

### 3.6. Alterations in the Gut Microbiota of *P. hominis*‐Infected Mice

To assess the impact of *P. hominis* infection on the gut microbiota in immunocompetent BALB/c mice, fecal samples were collected at 90 dpi from both infected and uninfected mice (*n* = 5 per group). Genomic DNA was extracted and subjected to 16 S rRNA amplicon sequencing, yielding a total of 3344 ASVs.

We first evaluated β‐diversity to compare the overall microbial community structure between the two groups. Principal coordinates analysis (PCoA) based on Bray‐Curtis distance revealed a clear separation between the infected control groups (PERMANOVA test; *p*  < 0.01), indicating a significant shift in gut microbiota composition following infection (Figure 6A). We next assessed α‐diversity to evaluate within sample diversity. Two indices were used: the Chao1 index (species richness) and the Shannon index (richness and evenness). Although the Chao1 index showed an increasing trend in the infected group, the difference was not statistically significant (Figure 6B). In contrast, the Shannon index was significantly higher in infected mice (*p*  < 0.05), suggesting that infection enhanced microbial diversity (Figure 6B).

Figure 6Alterations in gut microbiota following *P. hominis* infection. (A) Beta diversity analysis. Principal coordinates analysis (PCoA) plot based on Bray‐Curtis distances, illustrating beta diversity of gut microbiota in *P.hominis*‐infected and control mice. Each point represents ASVs from one sample (*n* = 5 per group). (B) Alpha diversity analysis. Comparison of microbial richness (Chao1 index) and diversity (Shannon index) between control and *P. hominis*‐infected mice.. (C–D) Microbiota composition at different taxonomic levels. Relative abundance of the top 10 phyla (C) and top 20 genera (D) in fecal samples from control and *P. hominis*‐infected mice. (E–F) Differential abundance analysis. Comparison of the relative abundance of selected bacterial phyla (E) and genera (F) between control and *P. hominis*‐infected groups. (G) Phylogenetic profiling. Cladogram illustrating the phylogenetic distribution of the bacterial lineages between control and *P. hominis*‐infected groups. Data shown as mean ± SEM (*n* = 5). Statistical analysis was determined by two‐tailed Student’s *t*‐test (B) or Fisher’s exact test (E,F). Statistical significance is indicated by  ^∗^ *p* < 0.05,  ^∗∗^ *p* < 0.01,  ^∗∗∗^ *p* < 0.001; ns, not significant.

**Figure:**
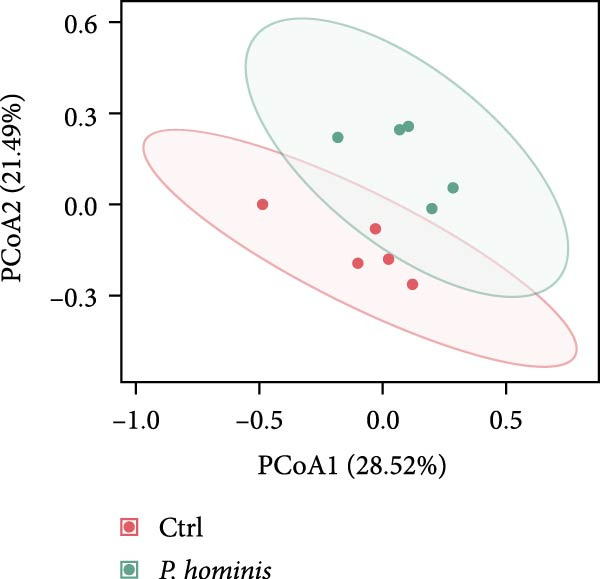
(A)

**Figure:**
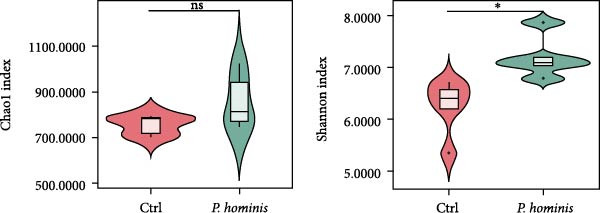
(B)

**Figure:**
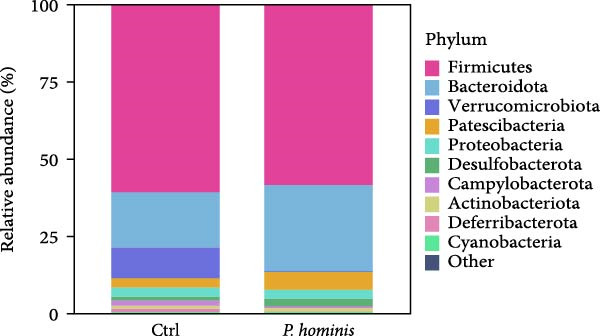
(C)

**Figure:**
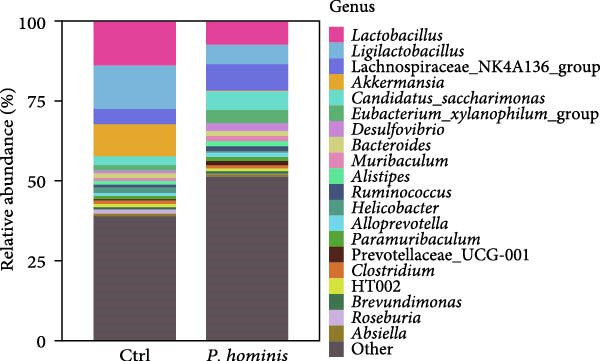
(D)

**Figure:**
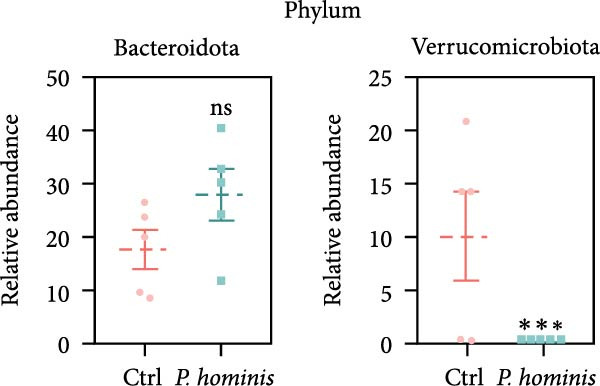
(E)

**Figure:**
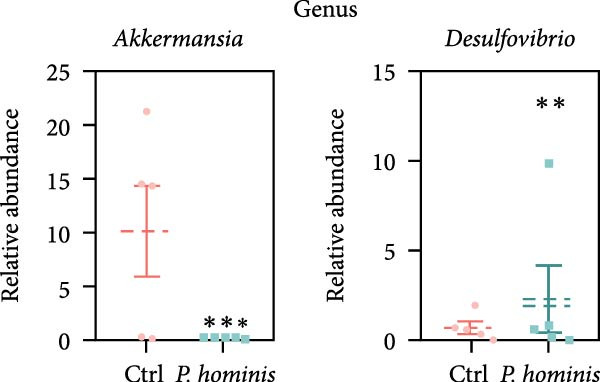
(F)

**Figure:**
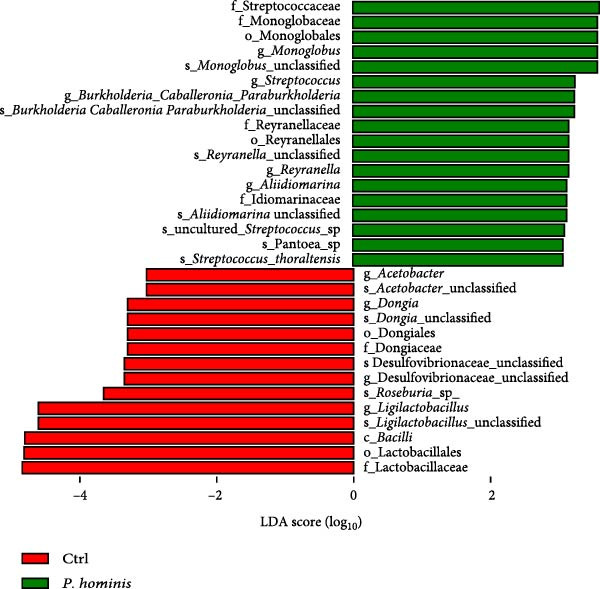
(G)

To further characterize compositional changes, we compared the relative abundance of bacterial taxa at different taxonomic levels. Community bar plots illustrated clear structural differences between the infected and control groups (Figure 6C,D). At the phylum level, the control group was dominated by Firmicutes, followed by Bacteroidota, Verrucomicrobiota, Patescibacteria, Proteobacteria, Campylobacterota, Desulfobacterota, Deferribacterota, Actinobacteriota, and Cyanobacteria (Figure 6C). In the infected group, the most notable changes included a nonsignificant increase in Bacteroidota (from ~17% to > 27%) and a sharp decrease in Verrucomicrobiota (from ~10% to < 1%; *p*  < 0.001; Figure 6C,E). Additionally, the Firmicutes/Bacteroidota ratio was lower in infected mice (Figure S6A). The relative abundance of Desulfobacterota increased significantly (*p*  < 0.05), while that of Campylobacterota decreased (*p*  < 0.05; Figure S6B). At the genus level, infected mice showed a marked increase in *Desulfovibrio* (*p*  < 0.01) and a pronounced reduction in *Akkermansia* (*p*  < 0.001; Figure 6D,F and Figure S6C). Other changes included decreases in *Lactobacillus, Ligilactobacillus, Helicobacte*r (*p*  < 0.05), and *Roseburia* (*p*  < 0.001), along with nonsignificant increases in Lachnospiraceae NK4A136 group, *Candidatus Saccharimonas*, and (*Eubacterium*) *xylanophilum* group (*p*  > 0.05; Figure S6C).

To identify potential microbial biomarkers, we performed linear discriminant analysis effect size (LEfSe) analysis. Several genera were enriched in the infected group, including *Monoglobus, Streptococcus, Burkholderia–Caballeronia–Paraburkholderia, Reyranella, and Aliidiomarina* (Figure 6G).

In summary, these findings demonstrate that *P. hominis* infection alters the gut microbiota community in immunocompetent mice, increasing its overall diversity while notably reducing beneficial genera, such as *Akkermansia* and *Roseburia*, and enriching inflammation‐associated taxa such as *Desulfovibrio*.

## 4. Discussion

*P. hominis* (formerly *Trichomonas hominis*), first identified by Davaine in 1854 [7], remains an understudied intestinal protozoan. Over a century after its discovery, fundamental aspects of its biology—including its life cycle, transmission routes, primary host, and animal reservoirs—are still poorly understood [11]. Crucially, its pathogenicity is still debated [12]. Although some evidence suggests it is pathogenic [13], such as an in vitro study showing that the *P. hominis* PHGD strain causes morphological alterations, reduced viability, and inflammatory responses in porcine intestinal (IPEC‐J2) cells [19], direct in vivo evidence has been limited. Our study now provides direct in vivo evidence that *P. hominis* infection induces significant and sustained pathology in the large intestine of healthy immunocompetent mice, characterized by inflammatory cell infiltration, epithelial necrosis, and mucosal erosion. These findings provide compelling support that *P. hominis* is a pathogenic protozoan capable of inducing chronic intestinal inflammation. However, it should be noted that our model employed a single high‐dose infection, which differs from natural settings where infections typically involve low‐level and repeated exposure to trophozoites. Additionally, the pathogenicity of *P. hominis* is likely to vary across host species, and research on its transmission and cross‐species potential is currently lacking. Thus, while this study demonstrates clear pathogenicity in mice, its precise impact on humans and other animals requires further investigation.

The intestinal tract constantly encounters a variety of potentially harmful agents present within the luminal contents; thus, it relies on multiple layers of barriers to defend against foreign antigens, pathogens, and gut microbiota [35]. The outermost luminal barrier consists of secreted mucus, which is primarily produced by goblet cells [36]. Impairment of the mucus barrier and alterations in goblet cell function have been associated with the development of chronic inflammation [33]. Consistent with the ability of *P. hominis* to induce chronic intestinal inflammation, we observed a marked increase in PAS staining along with a decrease in MUC2 levels in the large intestine following *P. hominis* infection. This apparent paradox may reflect impaired goblet cell maturation or dysregulated mucin secretion.

Chronic inflammatory disorders of the gastrointestinal tract, collectively referred to IBD, represent a significant global healthcare burden [35–37]. Although substantial efforts have been made to elucidate the pathogenesis of IBD, its etiology remains unclear. Pathogenic infections have been proposed as potential triggers for IBD, yet no specific pathogen has been definitively identified [38]. In our study, mice infected with *P. hominis* exhibited loose stools, intestinal inflammation, reduced body weight, and shorter intestinal length compared with the control group‐characteristics similar to those observed in DSS‐induced IBD mouse models [39]. Furthermore, we found that *P. hominis* infection triggered extensive infiltration of neutrophils into the lamina propria and muscularis mucosae of the large intestine, a hallmark feature of IBD [39]. Therefore, it is reasonable to speculate that *P. hominis* infection may be a risk factor for IBD. Further studies are needed to validate the clinical relevance of *P. hominis* infection in human IBD.

We have previously established a close association between *P. hominis* infection and the incidence of CRC [10]. However, it remains unclear whether the parasite actively contributes to the initiation or progression of CRC, or whether, conversely, immunocompromised cancer patients are more susceptible to *P. hominis* infection. Given that chronic intestinal inflammation can promote the proliferation and clonal expansion of transformed tumor cells and thereby drive tumor progression, we hypothesize that *P. hominis* may facilitate colorectal carcinogenesis through the induction of chronic inflammation [40–42]. Nevertheless, the precise role of *P. hominis* in CRC requires further clinical validation.

We unexpectedly observed that infection with *P. hominis* induced alveolar septal thickening in healthy immunocompetent mice. Although this parasite has been detected in human intrathoracic specimens [17, 43] and linked to pulmonary trichomoniasis [11], its microaerophilic nature raises doubt regarding its potential as a primary causative agent. Since alveolar septal thickening represents an early histopathological indicator of interstitial pneumonia, it is reasonable to hypothesize that *P. hominis* may contribute to pulmonary inflammation. The parasite may access the respiratory system through aspiration, possibly acting as an opportunistic pathogen that aggravates clinical symptoms and prolongs illness [11, 44]. Furthermore, accumulating evidence suggests that gut microbiota and their metabolites can influence lung immunity and function through the gut–lung axis [45, 46], suggesting a potential alternative pathway through which *P. hominis* infection could lead to pulmonary impairment. Further studies are warranted to clarify whether *P. hominis* directly colonizes the respiratory tract or exerts its effects through indirect mechanisms, and to establish its clinical relevance to interstitial pneumonia.

The gastrointestinal tracts of mammals harbor a complex microbial ecosystem comprising bacteria, protozoa, and viruses [20]. Interactions between intestinal protozoa and bacterial communities can directly or indirectly influence host health. Notably, intestinal disorders such as IBD and CRC have been linked to dysbiosis, characterized by altered composition and diversity of the gut microbiota [22]. In this study, we observed an increase in the relative abundance of Bacteroidetes and a decreased Firmicutes/Bacteroidetes (F/B) ratio following *P. hominis* infection—a trend consistent with findings reported in *P. hominis*‐infected female foxes [25]. Furthermore, a significant elevation in the abundance of *Desulfovibrio*, a genus with potential pathogenic traits, was detected in the infected group. *Desulfovibrio* has been implicated in gastrointestinal inflammation [47], and increased levels of *Desulfovibrio* have been observed in fecal samples from patients with UC [41, 42]. Based on these findings, *P. hominis* may promote chronic intestinal inflammation, at least in part, by facilitating the expansion of *Desulfovibrio*. We also observed that *P. hominis* infection reduced the relative abundance of potentially beneficial genera such as *Akkermansia*, which has been reported to be decreased in patients with IBD and is hypothesized to exert protective effects against colitis [48, 49]. Collectively, these results suggest that *P. hominis* induces chronic intestinal inflammation through specific shifts in gut microbiota composition—characterized by an increase in pro‐inflammatory bacteria and a decrease in beneficial taxa. Moreover, given the well‐established association between gut dysbiosis and colorectal tumorigenesis [21], it is plausible that the observed link between *P. hominis* infection and CRC may also be mediated by such microbiota alterations. Based on these findings, we propose that *P. hominis* may promote chronic intestinal inflammation, at least in part, by altering the gut microbiota composition—specifically through an increase in pro‐inflammatory bacteria and a decrease in beneficial taxa. However, it should be noted that while our study identifies significant microbial compositional changes following *P. hominis* infection, it does not include direct functional validation, particularly in terms of quantifying microbial‐derived metabolites. The observed reduction in major SCFA‐producing genera, including *Akkermansia*, *Roseburia*, and *Lactobacillus*, suggests a probable deficit in butyrate and other SCFAs, which are critical for maintaining colonic health through their roles in reinforcing the epithelial barrier and exerting anti‐inflammatory effects [50–52]. This potential decrease in SCFAs represents a plausible mechanistic link between *P. hominis*‐induced dysbiosis, and the ensuing chronic intestinal inflammation and barrier dysfunction. Therefore, direct measurement of SCFA levels in future studies will be essential to confirm this hypothesis and elucidate the underlying metabolic pathways.

While our study demonstrates chronic inflammation following *P. hominis* infection, the precise mechanistic drivers of this pathology remain to be elucidated. The epithelial injury could arise from several nonexclusive pathways: (1) direct cytotoxicity mediated by the physical interaction of the parasite with host cells; (2) the action of parasite‐secreted virulence factors such as proteases, which could degrade tight junction proteins and mucin, or pore‐forming toxins; or (3) immune‐driven collateral damage, where the host’s inflammatory response to the parasite (e.g., neutrophil infiltration) inadvertently damages the intestinal epithelium. Future studies aimed at directly addressing this question are warranted. Key experimental approaches such as applying transcriptomic (e.g., dual RNA‐seq) or proteomic analyses to the host‐parasite interface, either in vitro or from infected tissue samples, would provide a powerful, unbiased strategy to identify critical host signaling pathways and putative parasite virulence determinants, such as adhesion proteins and secreted hydrolases, that mediate the pathogenic outcome.

## 5. Conclusion

In conclusion, our study demonstrates that *P. hominis* infection induces chronic intestinal inflammation in healthy immunocompetent mice. These findings provide novel insights into the pathogenicity of this neglected intestinal protist and will facilitate further investigations into its pathogenic mechanisms.

## Conflicts of Interest

The authors declare no conflicts of interest.

## Author Contributions

**Yao Rong:** methodology, investigation, formal analysis, data curation. **Yidan Cheng:** methodology, investigation, formal analysis, data curation. **Hongbo Zhang:** investigation, data curation. **Xichen Zhang:** writing–review and editing, supervision. **Yu Zheng:** formal analysis, data curation. **Jianhua Li:**writing–review and editing, supervision. **Pengtao Gong:** writing–review and editing, supervision. **Xiaocen Wang:** formal analysis, data curation. **Xin Li:** formal analysis, data curation. **Nan Zhang:** writing–original draft, writing–review and editing, supervision, funding acquisition, conceptualization. Yao Rong and Yidan Cheng contributed equally to this work.

## Funding

This work was supported by the grants from the National Natural Science Foundation of China (Grant 32102696) and the National Key Research and Development Program of China (Grant 2021YFF0702900).

## Supporting Information

Additional supporting information can be found online in the Supporting Information section.

## Supporting information


**Supporting Information** Figure S1. Detection of *P. hominis* in stool samples of BALB/c mice by confocal microscopy and nested PCR at 7 dpi. (A) Confocal microscopy imaging. A representative confocal image showing *P. hominis* in the stool of a mouse inoculated with 1 × 10^6^ of trophozoites (refer to Figure 1A for parasite morphology). Scale bar: 5 μm. (B) Nested PCR detection. *P. hominis* was detected via nested PCR only in stool samples from mice inoculated with 1 × 10^6^ and 1 × 10^7^ of trophozoites. Lanes: M, 2000 bp DNA marker; 1, control group; 2, one trophozoite; 3, 10 trophozoites; 4, 1 × 10^2^ of trophozoites; 5, 1 × 10^3^ of trophozoites; 6, 1 × 10^4^ of trophozoites; 7, 1 × 10^5^ of trophozoites; 8, 1 × 10^6^ of trophozoites; 9, 1 × 10^7^ of trophozoites; 10, positive control. Figure S2. *P. hominis* infection induces pathological injury in the cecum and colon of BALB/c mice (related to Figure 1). Detailed histopathological features of the cecum and colon following *P. hominis* infection are shown. Left panels display low‐magnification images (40 × ) corresponding to those in Figure 1C; right panels show higher‐magnification views of the boxed areas. Scale bars: 200 μm. Figure S3. *P. hominis* infection induces long‐term effects in the large intestine over 90 days (related to Figure 2). Low magnification (40 × ) images of H&E‐stained large intestinal tissue sections from the indicated mouse groups were shown, corresponding to the regions presented in Figure 2D. Scale bars: 200 μm. Figure S4. No significant pathological or morphological changes were observed in major organs of BALB/c mice following *P. hominis* infection. (A) Histopathological analysis. Representative H&E‐stained sections of heart, liver, spleen, lung, and kidney tissues from infected and uninfected mice. Scale bars: 100 μm. (B) Macroscopic morphology. Representative photographs showing the appearance of heart, liver, spleen, lung, and kidney from infected and uninfected mice. Figure S5. *P. hominis* infection affects goblet cells and MUC2 expression (related to Figure 4). (A) PAS staining of goblet cells. Representative images of PAS‐stained sections showing goblet cells in cecal and colonic tissues from the indicated mouse groups at 90 dpi. Scale bars: 100 μm. (B) MUC2 immunohistochemical staining. Representative images of MUC2‐stained sections from cecal and colonic tissues of the indicated mice at 90 dpi. Arrows indicate inflammatory cell infiltration. Scale bars: 100 μm. Figure S6. Alterations in gut microbiome composition following *P. hominis* infection. (A) Firmicutes/Bacteroidetes ratio. Relative abundance ratio of Firmicutes to Bacteroidetes in fecal samples from control and infected mice. (B–C) Microbial composition at different taxonomic levels. Relative abundance of the top 10 phyla (B) and top 20 genera (C) in fecal samples from control and infected mice. Data are presented as mean ± SEM (*n* = 5). Statistical significance was determined by Fisher’s exact test ( ^∗^ *p* < 0.05,  ^∗∗^ *p* < 0.01,  ^∗∗∗^ *p* < 0.001; ns, not significant).

## Data Availability

16S rRNA sequencing data generated during this study are available in the National Center for Biotechnology Information (NCBI) BioProject Repository (https://www.ncbi.nlm.nih.gov/bioproject; Accession Number PRJNA1160716).
