# Supplementary material for: Pentatrichomonas hominis Infection Induces Chronic Intestinal Inflammation in Immunocompetent Mice
Source: Transbound Emerg Dis. 2025 Dec 13;2025:8820836. doi: 10.1155/tbed/8820836 (PMC12701619; doi:10.1155/tbed/8820836)
Supplement: Supplementary file 1 — Supporting Information Figure S1. Detection of P. hominis in stool samples of BALB/c mice by confocal microscopy and nested PCR at 7 dpi. (A) Confocal microscopy imaging. A representative confocal image showing P. hominis in the stool of a mouse inoculated with 1 × 10^6^ of trophozoites (refer to Figure 1A for parasite morphology). Scale bar: 5 μm. (B) Nested PCR detection. P. hominis was detected via nested PCR only in stool samples from mice inoculated with 1 × 10^6^ and 1 × 10^7^ of trophozoites. Lanes: M, 2000 bp DNA marker; 1, control group; 2, one trophozoite; 3, 10 trophozoites; 4, 1 × 10^2^ of trophozoites; 5, 1 × 10^3^ of trophozoites; 6, 1 × 10^4^ of trophozoites; 7, 1 × 10^5^ of trophozoites; 8, 1 × 10^6^ of trophozoites; 9, 1 × 10^7^ of trophozoites; 10, positive control. Figure S2. P. hominis infection induces pathological injury in the cecum and colon of BALB/c mice (related to Figure 1). Detailed histopathological features of the cecum and colon following P. hominis infection are shown. Left panels display low‐magnification images (40 × ) corresponding to those in Figure 1C; right panels show higher‐magnification views of the boxed areas. Scale bars: 200 μm. Figure S3. P. hominis infection induces long‐term effects in the large intestine over 90 days (related to Figure 2). Low magnification (40 × ) images of H&E‐stained large intestinal tissue sections from the indicated mouse groups were shown, corresponding to the regions presented in Figure 2D. Scale bars: 200 μm. Figure S4. No significant pathological or morphological changes were observed in major organs of BALB/c mice following P. hominis infection. (A) Histopathological analysis. Representative H&E‐stained sections of heart, liver, spleen, lung, and kidney tissues from infected and uninfected mice. Scale bars: 100 μm. (B) Macroscopic morphology. Representative photographs showing the appearance of heart, liver, spleen, lung, and kidney from infected and uninfected mice. Figure S5. P. hominis infection affects go [file TBED-2025-8820836-s001.docx]

**Supporting information**


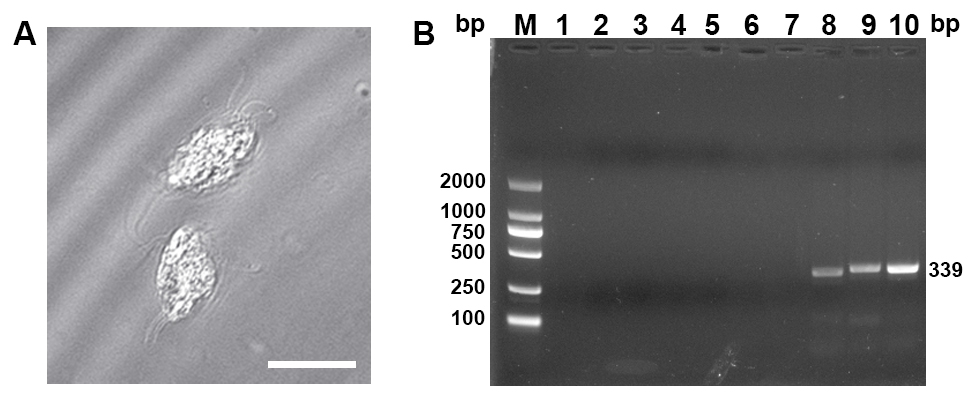


**Figure S1.** **Detection of *P. hominis* in stool samples of BALB/c mice by confocal microscopy and nested PCR at 7 dpi.**

1. Confocal microscopy imaging. A representative confocal image showing *P. hominis* in the stool of a mouse inoculated with 1×10^6^ of trophozoites (refer to Fig. 1A for parasite morphology). Scale bar: 5 μm.

(B) Nested PCR detection. *P. hominis* was detected via nested PCR only in stool samples from mice inoculated with 1×10^6^ and 1×10^7^ of trophozoites. Lanes: M, 2000 bp DNA marker; 1, control group; 2, one trophozoite; 3, 10 trophozoites; 4, 1×10^2^ of trophozoites; 5, 1×10^3^ of trophozoites; 6, 1×10^4^ of trophozoites; 7, 1×10^5^ of trophozoites; 8, 1×10^6^ of trophozoites; 9, 1×10^7^ of trophozoites; 10, positive control.


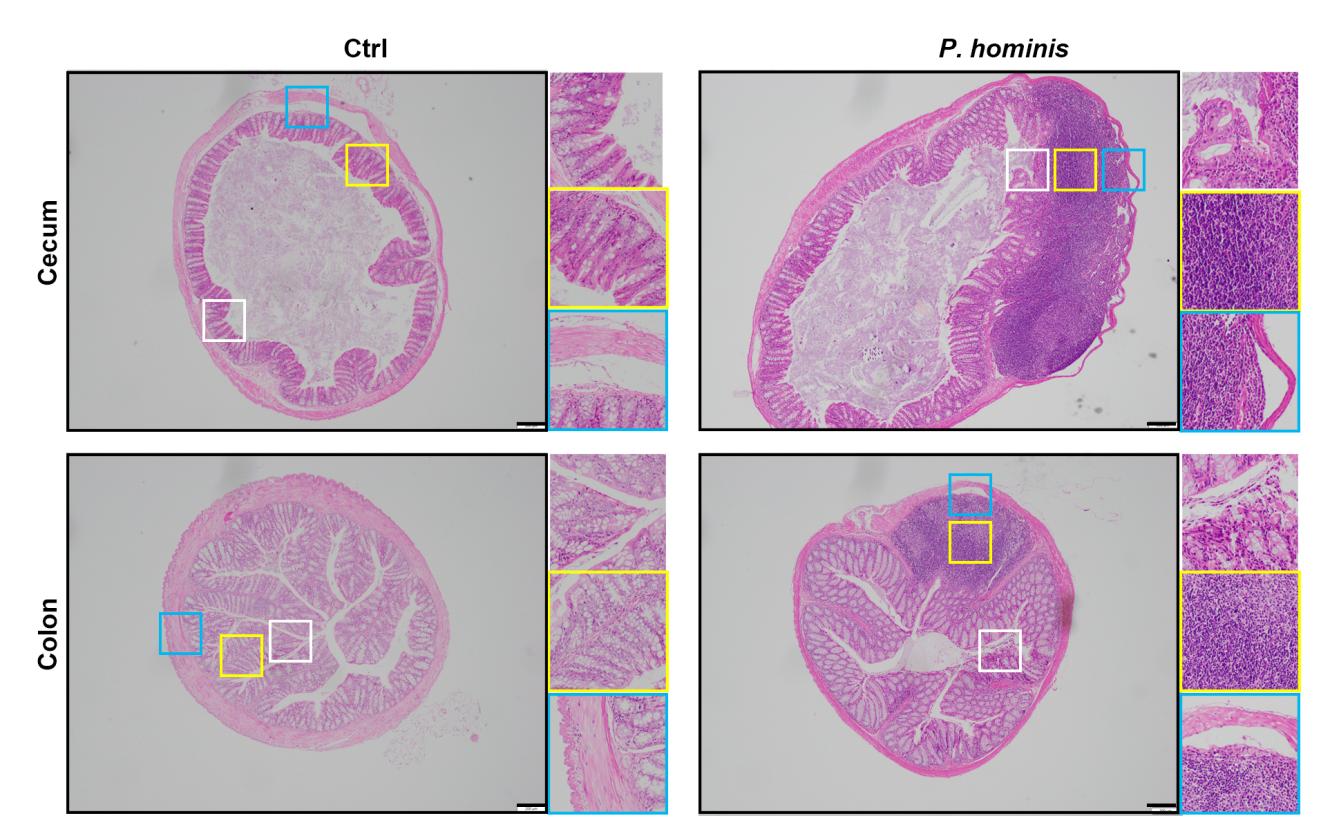


**Figure S2. *P. hominis* infection induces pathological injury in the cecum and colon of BALB/c mice (related to Fig. 1)**.

Detailed histopathological features of the cecum and colon following *P. hominis* infection are shown. Left panels display low-magnification images (40×) corresponding to those in Fig. 1C; right panels show higher-magnification views of the boxed areas. Scale bars: 200 μm.


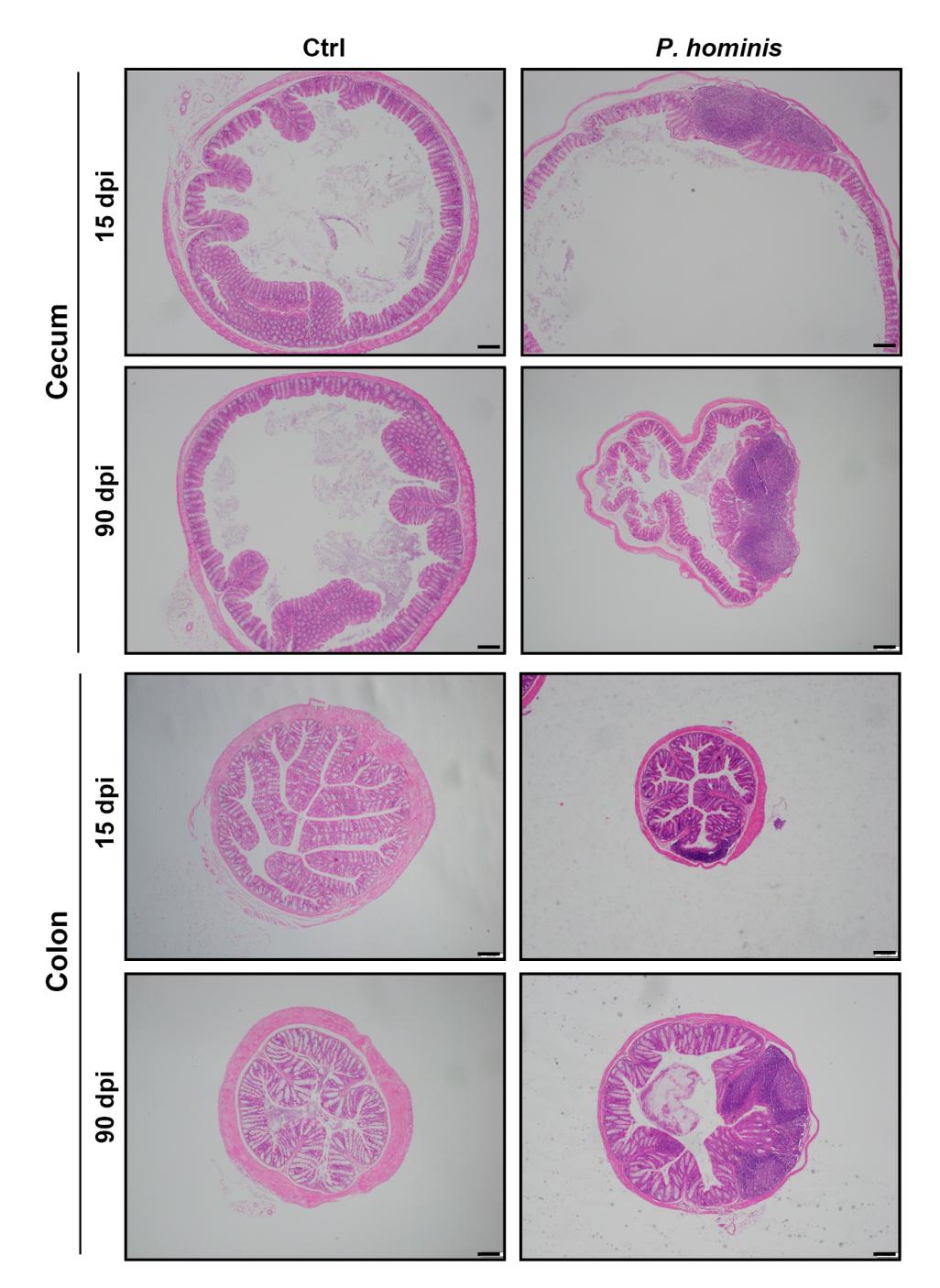


**Figure S3.** ***P. hominis* infection induces long-term effects in the large intestine over 90 days (related to Fig. 2)**.

Low magnification (40×) images of H&E-stained large intestinal tissue sections from the indicated mouse groups were shown, corresponding to the regions presented in Fig. 2 D. Scale bars: 200 μm.


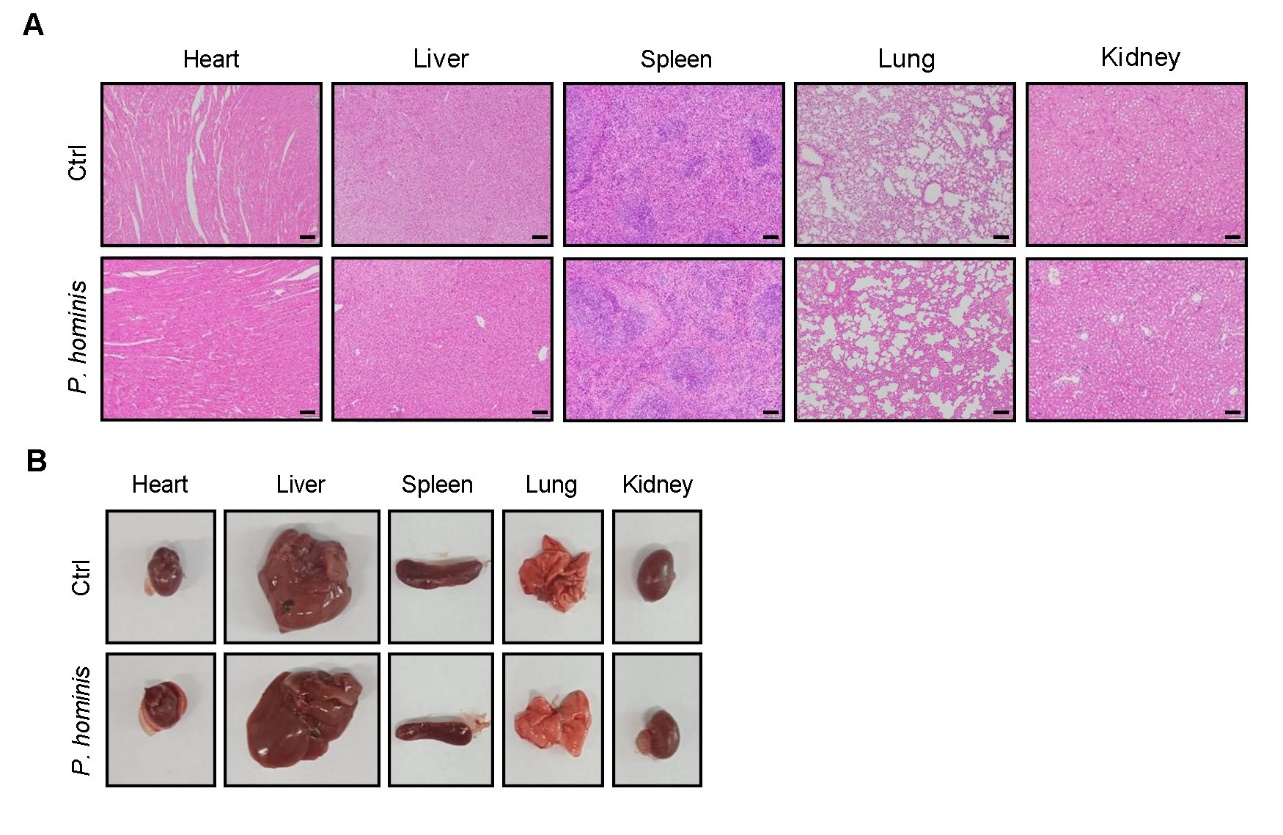


**Figure S4. No significant pathological or morphological changes were observed in major organs of BALB/c mice following *P. hominis* infection.**

1. Histopathological analysis. Representative H&E-stained sections of heart, liver, spleen, lung, and kidney tissues from infected and uninfected mice. Scale bars: 100 μm.

(B) Macroscopic morphology. Representative photographs showing the appearance of heart, liver, spleen, lung, and kidney from infected and uninfected mice.


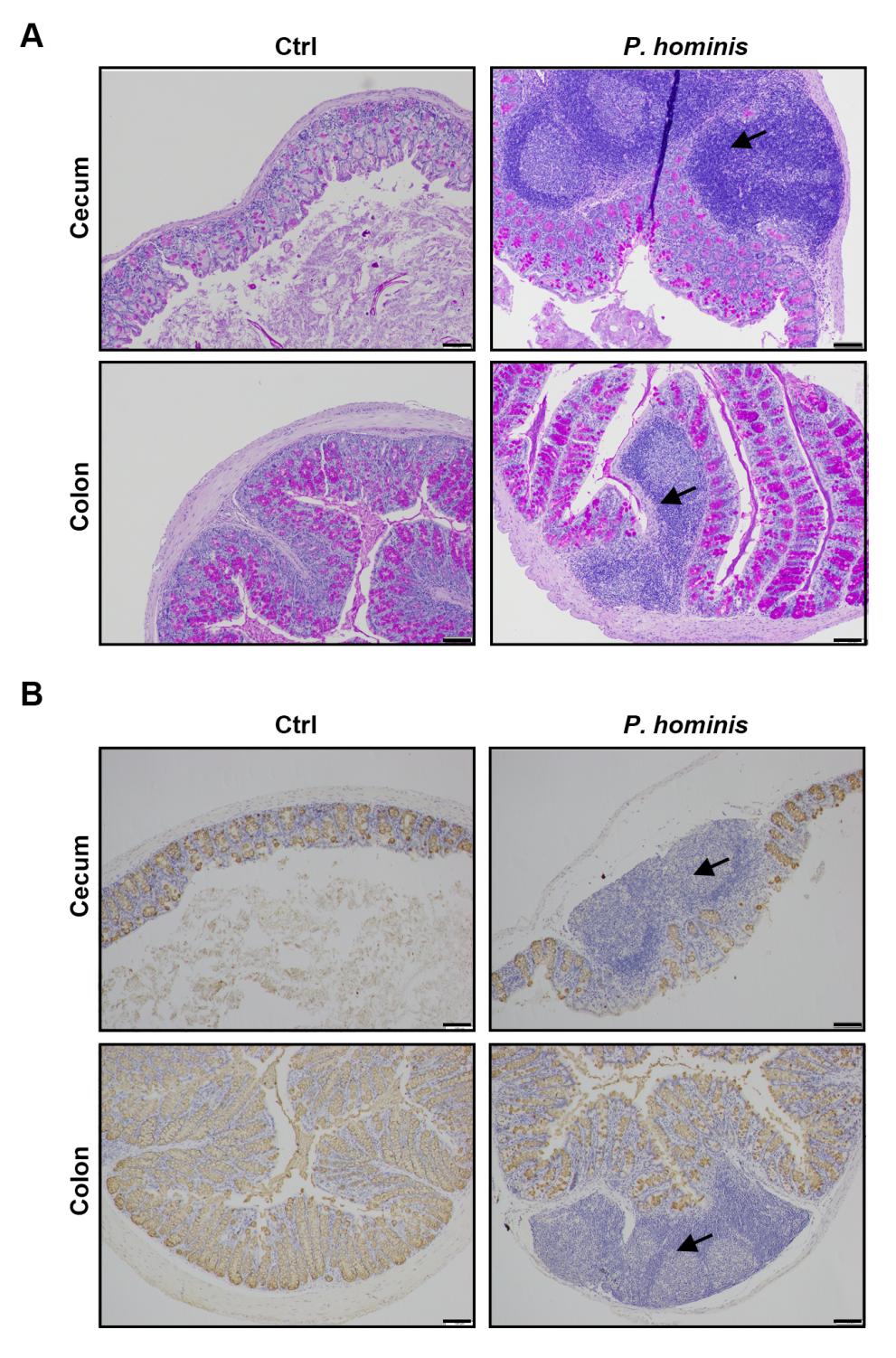


**Figure S5. *P. hominis* infection affects goblet cells and MUC2 expression (related to Fig. 4).**

(A) PAS staining of goblet cells. Representative images of PAS-stained sections showing goblet cells in cecal and colonic tissues from the indicated mouse groups at 90 dpi. Scale bars:100 μm.

(B) MUC2 immunohistochemical staining. Representative images of MUC2-stained sections from cecal and colonic tissues of the indicated mice at 90 dpi. Arrows indicate inflammatory cell infiltration. Scale bars: 100 μm.

**
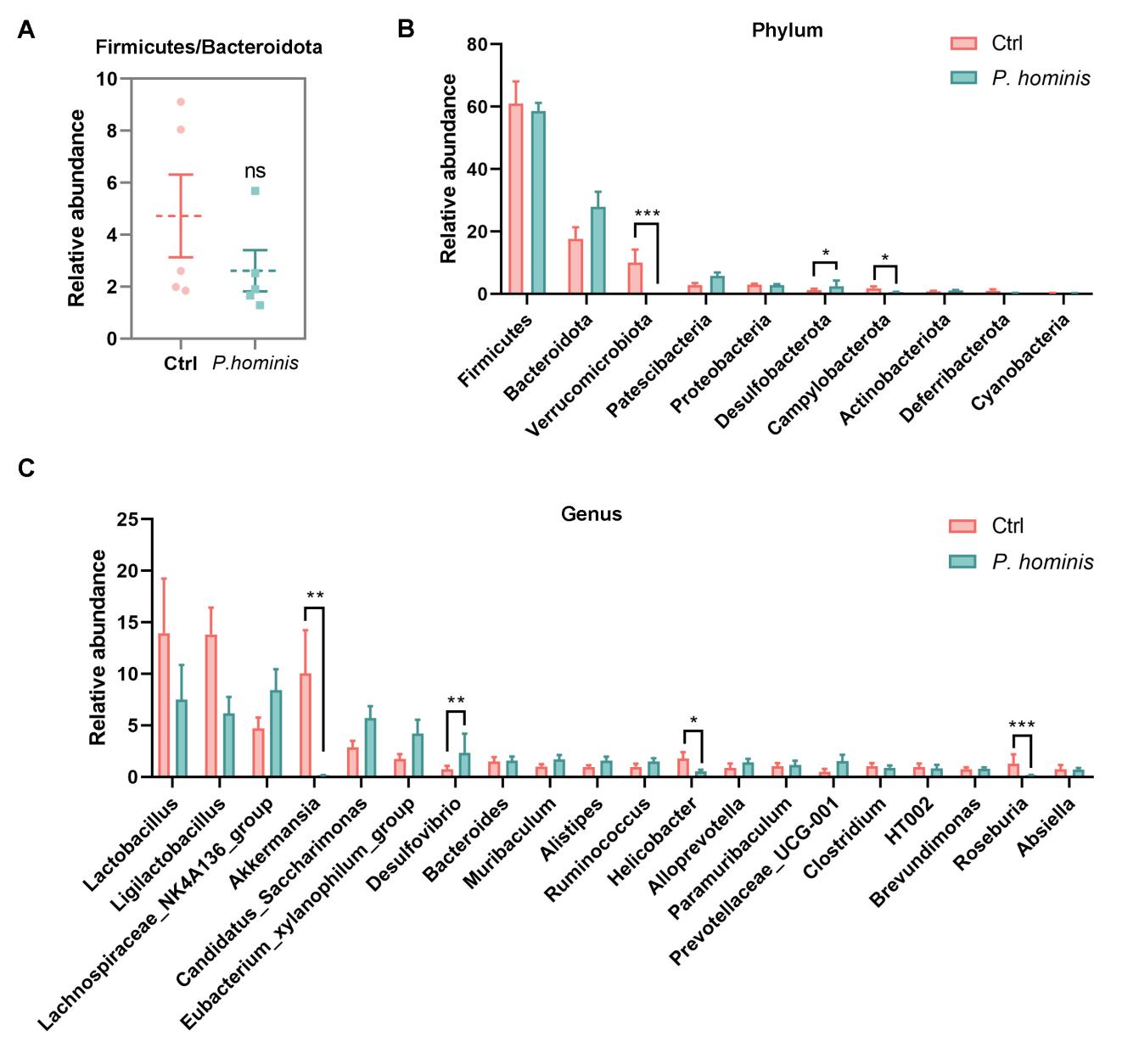
**

**Figure S6. Alterations in gut microbiome composition following *P. hominis* infection.**

1. Firmicutes/Bacteroidetes ratio. Relative abundance ratio of Firmicutes to Bacteroidetes in fecal samples from control and infected mice.
2. C) Microbial composition at different taxonomic levels. Relative abundance of the top 10 phyla (B) and top 20 genera (C) in fecal samples from control and infected mice.

Data are presented as mean ± SEM (n = 5). Statistical significance was determined by Fisher’s exact test (*p < 0.05, **p < 0.01, ***p < 0.001; ns, not significant).
